# Point of Care Sensing Devices: Better Care for Everyone †

**DOI:** 10.3390/s18124303

**Published:** 2018-12-06

**Authors:** Ajeet Kaushik, Mubarak A. Mujawar

**Affiliations:** 1 Center of Personalized Nanomedicine, Institute of NeuroImmune Pharmacology, Department of Immunology and Nano-Medicine, Herbert Wertheim College of Medicine, Florida International University (FIU), Miami, FL 33199, USA; 2 Department of Electrical and Computer Engineering, College of Engineering and Computing, Florida International University, Miami, FL 33174, USA

Improved health management is a key to provide a better health care [1,2,3]. Higher standards of health care management can be achieved by making a timely decision based on rapid diagnostics, smart data analysis, and informatics analysis. Thus, smart therapeutics, analytical tools, and diagnostics systems are always in demands to enhance the health wellness [4,5,6]. The optimization of a therapeutics is crucial to manage a disease, in terms of progression and monitoring evaluation, according to the patient profile. At the same time, disease progression/monitoring is vital for epidemic understanding and management. To reach this goal, it is imperative to conduct cutting edge research in order to design and develop smart diagnostic systems capable of performing as per patient profiles and make personalized health care possible [1,4,5,6,7].

Biosensors, smart analytical diagnostic tools, have been investigated with the potentials of point of care (POC) applications needed for personalized health care/management [8,9,10]. Keeping this in view, efforts are continuously being made to make biosensors more efficient, in terms of sensitivity, and of reduced form factors, in terms of portability [6,8,11]. Over the time, advancements in nanoscience and technology [1] have enabled biosensor development of desired salient features such as higher sensitivity, wider detection range, and low detection limits [11]. Smart electro-active functionalized nanostructures have also contributed to the development of biosensors by allowing for signal amplification, which is essential to achieve higher sensitivity and low detection limits of biosensor [11,12]. Emerging micro-electronics (e.g., miniaturized analyzer) and fabrication technologies (e.g., interdigitated electrodes) have also been useful to design and develop miniaturized and portable biosensors [13,14]. The integration of such biosensors with a BioMEMS technology has enabled automated and precise bio-sensing systems for better biological and clinical applications [15,16].

Recently, we have explored the self-assembled monolayer modified interdigitated microelectrodes to fabricate electrochemical cortisol (a psychological stress biomarker) biosensor (Figure 1A) [13]. This sensor detected cortisol at pM selectively in the saliva of farm-workers [17] and the plasma of the human immunodeficiency virus (HIV) positive patients [18]. This sensor was validated using an enzyme linked immunoabsorbent assay (ELISA) and was then further integrated with a miniaturized potentiostat to develop the sensor for POC application [15]. Similar kind of sensing prototype was also developed to detect the zika-virus protein at pM level [2,19,20]. Efforts are being made to use this sensor for real samples and compare the results with ELISA to optimize zika-virus protein detection for an early stage diagnostics at the site of the epidemic (Figure 1B) [2,21]. We have also explored the Electrochemical chip-based platform [22] to monitor the electrophysiology of cells infected with HIV-1 infection and treated with the anti-HIV drug (Figure 1C) [23,24]. Though such a developed chip needs a lot of optimization to present it as an alternative to expensive ELISA and polymerase chain reaction (PCR). In addition, efforts are being made to integrate the developed sensing platform with the smartphone to allow easy operation, systematic data management, and in time bio-informatics analysis for therapy decision.

The high selectivity and sensitivity of biosensors allow them to diagnose and manage targeted diseases at early stages and facilitate timely therapy decision. The advancements in the sensor integration and packaging technologies have enabled the disease diagnostics at POC [25]. The POC sensing systems are user-friendly and allow a much-needed diagnostics approach because of their easy operation, accessibility to disease sites and a rapid diagnostics in the remote areas where a suitable clinical laboratory set-up, and expertise is not accessible [1,2,26]. The artificial intelligence (AI) and the internet of things (IoT) have also been integrated with biosensors, allowing for real-time monitoring of biomarkers to generate bio-informatics needed for disease monitoring and to understand the epidemic and progression under therapy to optimize in time treatment [27,28].

In spite of significant advancements in the smart bio-sensing, efforts are continuously being made to develop cost-effective biosensors of reduced form factors for better health wellness. The implantable biosensor has been announced as the next generation biosensor for the personalized health care [29]. However, this objective has various obstacles, such as the fabrication of sensing units at the micro/nanoscale, bio-compatibility, implantation, sensor calibration, data sharing, and data analytics. Efforts are continuously being made to overcome these obstacles and by bringing chemist, physicist, biologist, electrical engineers, biomedical engineers, and clinicians at the same platform to explore best possible approaches.

Keeping advancements, challenges, and future prospects in mind, this special issue entitled “Point-of-Care Sensing Devices” for the MDPI-Sensors focuses on the ongoing research pertaining to the fundamental and applied research to develop biosensors for POC application. This special issue invites mini-reviews, reviews, letters, and research articles related to the field of nanotechnology, advanced functional sensing materials, numerical simulations, advancements in transduction, miniaturized sensing system development, AI and IoT. Technological advancement in signal amplification and developments in optical, electrical, magnetic, physical sensing strategies to fabricate an efficient sensing system is also covered in this issue.

This editorial encourages researchers to conduct active multidisciplinary and collaborative research oriented towards the design and development of smart POC sensing systems and to submit related research in the MDPI-Sensors journal. Editors would like to express sincere thanks to the contributors and reviewers for making this special issue successful and informative. We strive to make this special issue a value added to the scientific community by exploring nano-enabled sensing at POC for rapid and desired diagnostics needed for personalized health care and wellness.

## Figures and Tables

**Figure 1:**
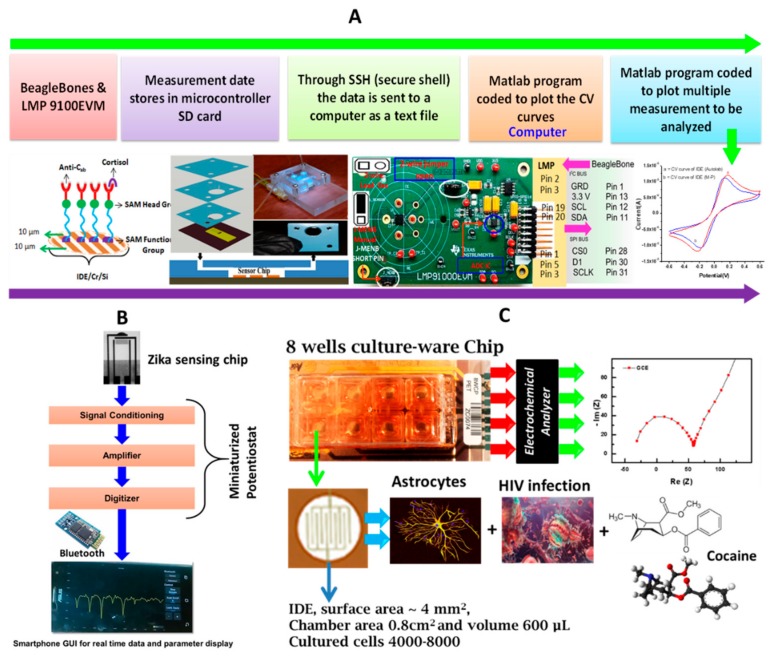
Illustration of the development of an electrochemical cortisol immune-sensor for POC application. The sensing system consists of a cortisol sensing chip integrated with a microfluidic manifold and interfaced with a miniaturized potentiostat (**A**) [15,18]. The electrochemical zika virus immunosensing chip for the detection of zika-virus envelope protein at 10 pM level (**B**) [2]. The chip-based electrochemical system to monitor the electrophysiology of cells during infection and treatment (**C**) [23,24].
